# Pulmonary Blastoma: A Rare Primary Lung Malignancy

**DOI:** 10.1155/2012/471613

**Published:** 2012-09-19

**Authors:** Mahmoud S. Alahwal, Iqbal H. Maniyar, Faiza Saleem, Mariam Alshiekh

**Affiliations:** ^1^Medical Oncology, Faculty of Medicine, King Abdulaziz University, Jeddah 22254, Saudi Arabia; ^2^Faculty of Medicine, King Abdulaziz University, Jeddah 22254, Saudi Arabia

## Abstract

Pulmonary blastoma, a rare primary lung malignancy, is considered to be distinct from other lung tumors based on pathological features, clinical course, and prognosis. More than one hundred cases have been reported in literature highlighting an interesting fact about their distinctive biologic manner from histopathological features. Classic pulmonary blastoma is composed of a mixture of immature epithelial and mesenchymal tissue resembling fetal lung tissue. Surgery is the mainstay of treatment. The prognosis of this rare malignancy is poor and the overall 5-year survival is around 15%. Our patient presented with respiratory symptoms and was found to have right-sided chest wall mass. The patient underwent complete surgical excision followed by 6 cycles of platinum-based chemotherapy. The patient showed good subjective and objective response with no evidence of disease recurrence. We report this rare malignancy with a review of literature, and the potential to use adjuvant chemotherapy in the management of this condition.

## 1. Introduction

Pulmonary blastoma (PB) is classified as one of the rare primary lung malignancies and is considered to be distinct from other lung tumors based on pathological features, clinical course and prognosis. PB is composed of a mixture of epithelial and mesenchymal tissues resembling embryonic lung tissue. Surgery is the standard treatment and the efficacy of adjuvant chemotherapy and radiotherapy has not yet been established. Platinum-based chemotherapy has been used with or without postoperative radiotherapy. The prognosis of this rare tumor is poor. We describe a case of this rare pulmonary malignancy and the role of adjuvant chemotherapy in the management and outcome.

## 2. Case Report

A 16-year-old young Egyptian female presented with progressive shortness of breath over a period of five years. The dyspnea was gradual in onset, at times precipitated by stress, but most of the time there was no precipitating factor. It was associated with severe nonproductive cough and bilateral headache. There was no significant past medical history. There was no history of fever, night sweats, and wheeze or chest pain. Also, there was no hoarseness of voice or hemoptysis. The patient sought medical advice at a private health center when the patient's daily activities gradually started getting affected. The patient was prescribed Salbutamol inhaler which gave her temporary relief. At the same time, the patient was diagnosed to have hyperthyroidism and was started on carbimazole. The patient took these medications for about 4 weeks and discontinued them against medical advice as she experienced lethargy and easy fatigability due to the sideeffects of the prescribed drugs. 

**Figure 1:**
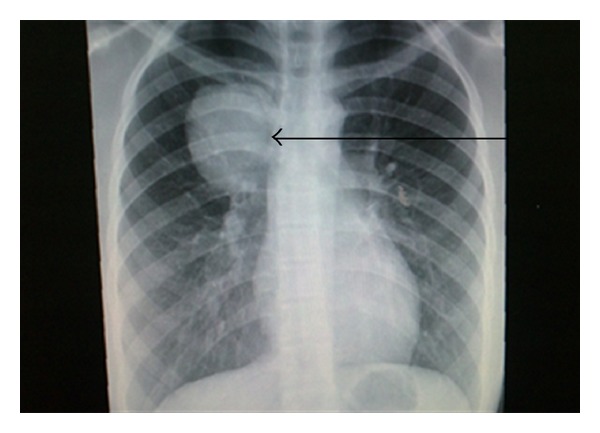
Posteroanterior radiograph of chest showing (arrow) right-sided circumscribed mass in the upper lobe close to the hilum.

**Figure 2:**
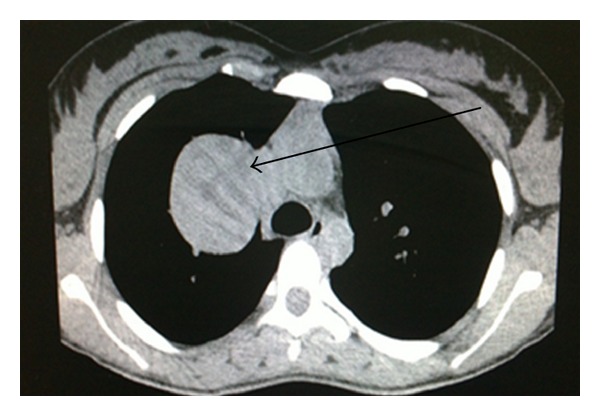
CT scan showing hypodensity (arrow) at the right upper lobe with significant compression to the superior venacava.

**Figure 3:**
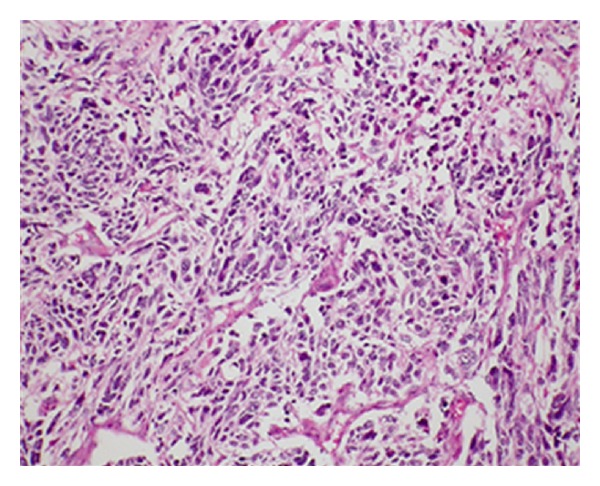
Histological features of the lesion containing epithelial and mesenchymal component consistent with pulmonary blastoma (3: HES ×100).

**Figure 4:**
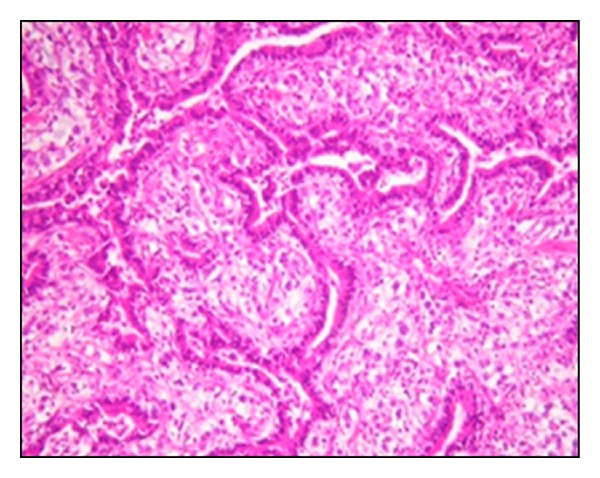
Histological features of the lesion containing epithelial and mesenchymal component consistent with pulmonary blastoma (H&E ×100).

After a brief period of relief of symptoms, the patient again started to have dyspnea and presented to the Cardiothoracic Unit in our hospital for evaluation. Baseline investigations were done, which included complete blood count, renal function tests, and liver function tests; all were within the normal range. Chest X-ray showed a well-demarcated right upper lobe mass close to hilum (Figure 1). Chest computed tomography (CT) scan showed bilateral thyroid nodules and a large well-defined heterogeneously enhancing mass measuring 11 × 6 cm with a central hypodensity at the right upper lobe with significant compression to the superior vena cava (Figure 2). There was no evidence of significant lymphadenopathy or pleural effusion. Complete surgical removal of the tumor along with safety margins was done and sent for histopathology examination. The biopsy report revealed ill-defined neoplastic growth showing biphasic pattern comprising epithelial and mesenchymal components consistent with pulmonary blastoma (Figures 3 and 4). The surgical margins were free of tumor. CT abdomen and bone scintigraphy showed no evidence of distant metastasis. The patient was referred to Radiation Oncology Department for possible intervention, but due to lack of established evidence of the role of radiotherapy in controlling local recurrence, the patient did not receive adjuvant radiotherapy. Due to the presence of certain poor prognostic factors like biphasic type and tumor size more than 5 cm, it was decided to proceed with adjuvant platinum-based chemotherapy comprising of ifosfamide, carboplatin, and VP-16 (ICE protocol). The patient received 6 cycles of the ICE protocol every 3 weeks with prophylactic granulocyte colony stimulating factor (G-CSF) from day 5 to day 12 after each cycle. There was one episode of febrile neutropenia after cycle 4 and the patient recovered completely after treatment with intravenous antibiotics. Reevaluation was done after 3rd and 6th cycle with CT chest which showed no evidence of recurrence. The patient is in complete remission and currently on a 3-month ongoing followup. 

## 3. Discussion

Pulmonary blastoma is a rare primary lung malignancy, comprising only 0.25–0.5% of all malignant lung neoplasm [1]. In 1952, Barnard described it as “lung embryoma” based on the histological resemblance of the tumor to fetal lung tissue [1]. The term blastoma was introduced later in 1961 by Spencer who described the tumor origin from a pleuripotential pulmonary blastoma analogous to Wilm's tumor that appears to share the same histological features [1]. 

More than one hundred cases have been reported in literature highlighting an interesting fact about their distinctive biologic manner from histopathological features [2]. PB is classified into 3 subtypes based on tissue component such as the monophasic PB, known as the well-differentiated fetal adenocarcinoma (WDFA) comprising epithelial malignant component only; the classic biphasic pulmonary blastoma (CBPB) characterized by both epithelial and mesenchymal malignant components; pleuropulmonary blastoma (PPB), that is, a childhood tumor showing features of mesenchymal malignant components only. CBPB is the most common of the three subtypes [2]. PB is observed in the fourth decade of life with mean age of occurrence in adults being 43 years, and shows a strong female preponderance [3]. Our patient, a previously healthy, young female, was experiencing typical symptoms like recurrent cough and shortness of breath for 5 years. The prolonged history should have alerted treating physicians of an underlying pathology. However, the non-specific symptoms might be attributed to the delay in arriving at a precise diagnosis. Initially, she was misdiagnosed as bronchial asthma and was prescribed Salbutamol inhaler which seem to alleviate the symptoms for a brief period of time. On presentation to our hospital, a simple non-invasive test led to a series of events confirming the diagnosis of CBPB. 

About 80% of CBPB occur in adults, and can occur at any age between birth and seventy years [4]. Unlike PB, CBPB shows a slight male preponderance and is common among smokers [4]. Interestingly, our patient was a teenager, nonsmoker female patient. Symptoms in CBPB are likely to seem like an upper respiratory infection, but recurrent, persistent symptoms require investigations. In CBPB, chest radiography is usually helpful and typically shows a well-demarcated peripheral or midlung mass with no definite lobar predominance [4]. In our case also, a well-circumscribed lesion was seen in the right middle lobe which was confirmed on chest CT scan showing a mixed solid and cystic lesion with variable contrast enhancement and a necrotic center with no evidence of involvement of pleura or mediastinum, which would have otherwise indicated metastasis.

Immunohistochemistry plays a vital role in the diagnosis of CBPB. Each component can be clearly distinguished with each other when a combination of both epithelial and mesenchymal markers is used [4]. In our case, complete excision biopsy was done, and a panel of immunohistochemical markers confirmed the biphasic pattern comprising epithelial and malignant mesenchymal components consistent with CBPB.

Surgical resection is the mainstay of treatment for the patients with PB. A number of cases of surgical approach with curative intent have been reported including an interesting case with a 24-year complete remission postsurgery [5]. 

Literature regarding the efficacy of adjuvant chemotherapy and radiotherapy is scarce. The vague role of chemotherapy in the adjuvant setting is probably due to rarity of this malignancy and seems to be individualized in reported cases. Various combinations of chemotherapeutic drugs have been tried as neoadjuvant and adjuvant treatment. However, several case reports document the use of platinum-based adjuvant treatment [3, 4]. 

Our patient received 6 cycles of the ICE protocol comprising of ifosfamide, carboplatin, and etoposide. Reevaluation was done by CT scan of chest after 3rd and 6th cycles of chemotherapy which showed no evidence of recurrence. The patient experienced acute emesis postchemotherapy during the first 3 cycles. Prophylactic granulocyte colony-stimulating factor (G-CSF) was given for 1 week from day 5 after each cycle. The patient had not been considered as a candidate for radiotherapy due to the presence of poor prognostic features, in addition to lack of sufficient data supporting the definite role of radiation in the adjuvant setting. The patient is on ongoing 3 monthly follow up with complete remission. Reevaluation CT scans will be done, and the patient is advised to seek immediate medical care in case of appearance of previous or novel symptoms.

 Although, several individual case reports of long-term survival have been documented, the overall prognosis of this rare malignancy is poor, and two-thirds of patients die within 2 years of diagnosis [5]. In one survey of 83 cases, the mean survival of approximately 33 months was reported for the patients who underwent surgery and achieved negative margins [5]. 

The factors contributing to the unfavorable prognosis are the biphasic type, tumor recurrence, metastatic disease on presentation, tumor size over 5 cm, and frequent lymph node involvement [1, 2].

In our case, the decision to start adjuvant treatment was based on the presence of two important prognostic factors, namely, the large size of the tumor on presentation (around 11 cm) and biphasic pattern of the disease. Despite not receiving any postoperative radiotherapy, the patient continues to be in remission until last followup. 

In conclusion, whether adjuvant treatment plays a role in the remission or not is a debatable issue, this case suggests a wait- and- watch approach when platinum-based chemotherapy is given as an adjuvant in the management of PB. Although a large series of case studies is required to further analyze and establish a framework for the definite role of adjuvant chemotherapy, and owing to the rarity of this malignancy, adjuvant management following surgery may be based on individual cases depending on the histopathology and stage of the tumor.
